# Cytotoxic activity of essential oil from Leaves of *Myrcia splendens* against A549 Lung Cancer cells

**DOI:** 10.1186/s12906-023-03969-y

**Published:** 2023-05-02

**Authors:** Monalisa Martins Montalvão, Franciel Batista Felix, Edmilson William Propheta dos Santos, Jileno Ferreira Santos, Waldecy de Lucca Júnior, Atenilton Santos Farias, Adauto de Souza Ribeiro, Carlos Cavaleiro, Samísia Maria Fernandes Machado, Ricardo Scher, Cristiane Bani Corrêa

**Affiliations:** 1 Department of Morphology, Federal University of Sergipe (UFS), São Cristovão, Sergipe Brazil; 2 Department of Morphology, Federal University of Minas Gerais (UFMG), Belo Horizonte, Minas Gerais Brazil; 3 Department of Chemistry, Federal University of Sergipe (UFS), São Cristovão, Sergipe Brazil; 4 Department of Ecology, Federal University of Sergipe (UFS), São Cristovão, Sergipe Brazil; 5 Faculty of Pharmacy, University of Coimbra, Coimbra, Portugal

**Keywords:** *Myrcia splendens*, Essential oil, Cytotoxicity, Apoptosis, Clonogenic assay, Cell migration

## Abstract

**Background:**

Plants of the *Myrcia* genus have been widely used in folk medicine to treat various diseases, including cancer. *Myrcia splendens* species has a diverse chemical constitution, but the biological activities of its essential oil have not been well investigated. In this study to out the chemistry characterization of essential oil (EO) from the leaves of the species *M. splendens* from Brazil and evaluate cytotoxic effect in A549 lung cancer cells.

**Methods:**

*M. splendens* EO was obtained by hydrodistillation and analyzed by Gas Chromatography-Mass Spectrometry (GC-MS). EO was isolated and evaluated for cellular viability in tumor cell lines by MTT assay. The evaluation of the formation of clones and the migratory capacity of the A549 cells treated with EO was done by the clonogenic assay and the wound healing assay. Morphological changes were observed in A549 cells by fluorescence using Phalloidin/FITC and DAPI.

**Results:**

22 compounds were identified in the chemical analysis of EO, corresponding to 88% of the sample. Major compounds were the sesquiterpenic hydrocarbons bicyclogermacrene (15.4%), germacrene D (8.9%) and E-caryophyllene (10.1%). The biological analysis of the EO showed high cytotoxic activity with an IC_50_ below 20 µg/ml in the THP-1, A549 and B16-F10 tumor cells. The treatment with EO reduced colony formation and inhibited the migratory capacity of A549 cells. Furthermore, apoptotic morphological changes in the nucleus and cytoplasm of A549 cells was observed after of treatment with EO.

**Conclusion:**

The findings of this study suggest that the *M. splendens* EO has cytotoxic compounds for the A549 lung cancer cells. Treatment with the EO decreased the colony formation and reduced the ability of lung cancer cells to migrate. Future studies may be used to isolate compounds from the EO for the study of lung cancer.

## Background

Cancer is a growing health problem in the world and there are still limitations in the treatment with chemotherapy, which in general cause several toxic effects [1]. The total number of cancer cases is expected to increase from 19.3 million in 2020 to 30.2 million in 2040, with nearly 10 million deaths worldwide accounted for in 2020 alone [2]. These data highlight the importance of using effective alternative and complementary treatments.

Recently there was growth in the studies of biological properties of natural products due its potential to treat several diseases, as cancer [3, 4]. Among these products, essential oils stand out, secondary metabolites that constitute a source of several bioactive compounds with anticancer potential, with their antitumor effects reported in in vitro and in vivo studies [5, 6]. In this regard, it is known that tropical plants have several essential oils with biological properties, as is the case with plants of the Myrtaceae family [7].

*Myrcia splendens* (SW.) DC. (Myrtaceae) is a tree plant with a wide distribution from Mexico to the South of Brazil [8]. In Brazil it is also known as “guamirim-da-folha-miúda” and in traditional medicine it is used to treat diarrhea, diabetes and hypertension [9, 10]. Some studies have demonstrated the biological properties of *M. splendens* EO including antibacterial [11], antifungal [12] and cytotoxic effects in tumor cells [9]. However, little is known about the antitumor activities of *M. splendens* EO. We hypothesize that EO has high in vitro antitumor activity. Thus, the present study aims to explore the antitumor effect of *M. splendens* EO on human tumor cell lines in vitro.

## Methods

### Plant material

Fresh leaves of *M. splendens* were collected in Ibura Flora, N. S. do Socorro, Sergipe, Brazil, and identified by Dr. Adauto de Souza Ribeiro, Federal University of Sergipe. The use of the leaves has been authorized by the Agency for Research in Federal Conservation Units (SISBIO) of the Chico Mendes Institute for Biodiversity Conservation (ICMBio) (collection authorization No. 68163/2019 – SISBIO – ICMBio). A voucher specimen (ASE 33399) is deposited at the Herbarium of the Department of Biology, Federal University of Sergipe, Brazil.

### Essential oil isolation

Plant material was coarsely divided submitted to hydrodistillation, during 3 h, using a modified Clevenger apparatus and the procedure described in the European Pharmacopoeia [12]. The essential oil was obtained with a yield of 0.3% (w/w), dried over anhydrous sodium sulfate and kept in the dark at 4ºC prior to use.

### GC/MS analysis

Composition of the oil was accessed by gas chromatography (GC) and gas chromatography-mass spectroscopy (GC/MS). Analytical GC was carried out in a Hewlett-Packard 6890 (Agilent Technologies, Palo Alto, CA, USA) gas chromatograph with HP GC Chem-Station Rev. A.05.04 data handling system, equipped with a single injector and two flame ionization detection (FID) systems. A graphpack divider (Agilent Technologies, part no. 5021–7148) was used for simultaneous sampling to two Supelco (Supelco, Bellefonte, PA, USA) fused silica capillary columns with different stationary phases: SPB-1 (polydimethylsiloxane 30 m x 0.20 mm i.d., film thickness 0.20 μm) and Supelcowax-10 (polyethylene glycol 30 m × 0.20 mm i.d., film thickness 0.20 μm). Oven temperature program is 70–220 °C (3 °C.min^− 1^), 220 °C (15 min), with injector temperature: 250 °C, carrier gas: helium, adjusted to a linear velocity of 30 cm.s^− 1^; splitting ratio 1:40; detectors temperature: 250 °C. GC-MS was carried out in a Hewlett-Packard 6890 gas chromatograph fitted with an HP1-fused silica column (polydimethylsiloxane 30 m × 0.25 mm i.d., 0.25 μm film thickness) interfaced with a Hewlett-Packard 5973 mass selective detector (Agilent Technologies) operated by HP Enhanced ChemStation software, version A.03.00. GC parameters are described above, with interface temperature: 250 °C, MS source temperature: 230 °C, MS quadrupole temperature: 150 °C, ionization energy: 70 eV, ionization current: 60 mA, scan range: 35–350 units, and scans.s^− 1^: 4.51. Essential oil components were identified by their retention indices on both SPB-1 and Supelcowax-10 columns and from their mass spectra. Retention indices, calculated by linear interpolation to retention times of C8–C23 n-alkanes, were compared with those of reference samples included in the Faculty of Pharmacy, University of Coimbra database. Acquired mass spectra were compared with reference spectra from laboratory database, Wiley/NIST library and literature data [13]. Relative amounts of individual components were calculated based on GC raw data areas without FID response factor correction.

### Cells

The cytotoxicity of the EO was tested against lung adenocarcinoma (A549), melanoma (B16-F10) and acute monocytic leukemia (THP-1) cancer cell lines, all obtained from American Type Culture Collection (ATCC). Cells were grown in Roswell Park Memorial Institute (RPMI) 1640 medium or in Dulbecco’s Modified Eagle’s Medium (DMEM) supplemented with heat-inactivated Fetal Bovine Serum (FBS, 10%) and 100 U/mL penicillin with 100 µg/mL streptomycin, and incubated at 37 °C with a 5% CO_2_ atmosphere.

### MTT assay

The cytotoxicity activity was quantified using the MTT (3 - [4, 5-dimethylthiazole-2-yl]-2, 5-diphenyl-tetrazolium bromide) assay, as previously described by [14]. For all experiments, cells were seeded in 96-well plates (2 × 10^4^ cells/mL in 200 µL of medium) in incubated overnight to permit attachment. After 24 h, the EO (2,5–100 µg/mL), dissolved in Dimethylsulfoxide (DMSO) or Doxorubicin (25 µg/mL) was added to each well and incubated for 24 h. Subsequently, 200 µL of MTT (5 mg/mL in PBS) was added to each well and further incubated for 3 h later, the formazan product was dissolved in 150 µL of DMSO, and the absorbance was read in a microplate reader (Synergy H1, Biotek, VT, EUA) at 570 nm. Cytotoxicity was expressed as the concentration of oil inhibiting cell viability by 50% (IC_50_). All measurements were performed in triplicate and the means and standard errors were calculated.

### Clonogenic assay

The procedure of Franken et al. (2006) was employed with some modifications [15]. A549 cells were seeded in 6-well plate (300 cells/well) in RPMI medium containing 10% FBS and 1% antibiotic (penicillin 10,000 U/ml; streptomycin 10,000 mg/ml). After 24 h of incubation conditions in an oven with an atmosphere of 5% CO_2_ at 37 °C, the cells were treated with EO at concentrations of 10, 20 and 40 µg/mL, which correspond to the values of 0.5xIC_50_, 1xIC_50_ and 2xIC_50_, respectively. DMSO 0.1% and Doxorubicin 25 µg/mL were used as a negative control and a positive death control, respectively. After the treatment time, the media were removed and complete DMEM medium was added to the wells, and the cells were incubated in an oven with a 5% CO_2_ atmosphere at 37 ºC for 10 days. After aqueous solution with acid + water (3:5 min) stained with crystal violet, 5% in 30 min. At the end of the experiment, the growth pattern in number of colonies of cells was observed and counted, with the aid of Image J software.

### Wound healing assay

A549 cells were seeded in 12-well plate at a density of 3 × 10^5^ cells per well. After 24 h, the cell monolayer was scratched with a tip of p200 pipette creating a straight-line wound, the debris were removed by washing with PBS and the cells were treated with 10, 20 and 40 µg/mL the EO for 24 h. DMSO 0.1% was used as vehicle control and Doxorubicin 10 µM was used as positive control. The images were acquired 0, 24 and 48 h of the scratch using a microscopy Olympus. The percentage of wound closure was calculated for each treatment and controls comparing the time points 24 and 48 h with the time point zero, using the equation proposed by Yarrow et al. (2004) [16]. The images were analyzed using ImageJ 1.46 software.

### Cell morphology assay

A549 cells were seeded at a concentration of 1 × 10^4^ in 48-well plates, in RPMI medium containing 10% FBS and 1% antibiotic (penicillin 10,000 U/mL; streptomycin 10,000 mg/mL). After 24 h of incubation, the cells were exposed to concentrations of 10, 20 and 40 µg/mL the EO and incubated for 24 h in an oven. DMSO 0.1% and Doxorubicin 25 µg/mL were used as negative control and positive control, respectively. Culture medium from all wells of the plate was removed and the cells washed three times with 1X PBS. After washing, cells were fixed with 4% formaldehyde at room temperature for 15 min and then washed again with 1X PBS. Cells were permeabilized using 0.2% Triton X-100 solution diluted in PBS for 15 min and then 1% Bovine Serum Albumin (BSA) also diluted in PBS was used for 30 min. To visualize the cytoskeleton, cells were stained with Phalloidin/FITC (25 µg/mL) for 30 min, in the dark, followed by 2 washes with PBS. The cell nucleus was counterstained with DAPI (1 µg/mL) for 10 min, in the absence of light. Images were captured at 200x and 400x magnification under a fluorescence microscope (Olympus, USA).

### Statistical analysis

For all experiments, a 95% confidence interval was used and p < 0.05 values considered statistically significant. Analyzes and graphs as well as IC_50_ were obtained using the GraphPad Prism 8. Shapiro-Wilk normality test was applied to assess the normal distribution of the data. For comparison between groups, ANOVA was used, followed by Dunnett post-test.

## Results

### Chemical composition of the *M. splendens* essential oil

The analysis of the essential oil from the leaves of *M. splendens* resulted in the identification of 22 constituents that represent 88% of the total composition (Table 1). The oil is composed of hydrocarbon sesquiterpenes (48.5%) and oxygen containing sesquiterpenes (39.6%), being bicyclogermacrene (15.4%), E-caryophyllene (10.1%) and germacrene D (8.9%) the major constituents.

**Table 1:** Composition of the essential oil from the leaves of *M. splendens*

RI ^a^	RI ^b^	Compounds*	Relative amount in sample (%)
			MS
1329	1467	δ-Elemene	2.9
1342	1455	α-Cubebene	0.5
1369	1487	*α-*Copaene	2.2
1380	1536	*β*-Cubebene	0.7
1376	1517	*β*-Bourbonene	1.7
1408	1590	*E-*Caryophyllene	10.1
1422	2130	*γ*-Elemene	0.5
1427	1600	Aromadendrene	0.3
1442	1662	*α*-Humulene	2.4
1466	1699	Germacrene D	8.9
1470	1715	*β*-Selinene	0.2
1479	1726	Bicyclogermacrene	15.4
1508	1751	*δ*-Cadinene	2.7
1526	2070	Elemol	7.5
1553	2113	Spathulenol	1.6
1557	1975	Caryophyllene oxide	0.6
1562	2063	Globulol	0.6
1576	2079	Guaiol	7.7
1607	2453	*γ*-Eudesmol	3.7
1622	2215	*β*-Eudesmol	5.0
1628	2208	*α*-Eudesmol	5.1
1639	2196	Bulnesol	7.8
Sesquiterpene hydrocarbons			48.5
Oxygen containing sesquiterpenes			39.6
**Total identified**			**88.0**

(*) - Compounds listed in order of their elution on the SPB-1 column. RI^a^ - Retention index determined on the non-polar SPB-1 column relative to a series of n-alkanes (C8–C23). RI^b^ - Retention index determined on a SupelcoWax-10 column relative to a series of n-alkanes (C8–C23)

### *M. splendens* essential oil reduce the viability of cancer cell lines

In order to assess the effects of EO on the viability of cancer cell lines, after the treatment of *M. splendens* EO at varied concentrations for 24 h, the cytotoxic activities were evaluated in three tumor cell lines: A549 (lung cancer), THP-1 (acute monocytic leukemia) and B16-F10 (melanoma), using the MTT assay. IC_50_ values and confidence interval are shown in Table 2. EO was cytotoxic in all tested tumor lines, presenting IC_50_ values of 5.37 µg/mL (2.17–13.30) in THP-1 cells, of 17.76 µg/mL (9.48–33 0.26) in B16-F10 cells and 20.14 µg/mL (16.46–24.64) in the A549 cells. Doxorubicin was used as a positive control and showed an IC_50_ value of 25.51 µg/mL in A549 cells. Considering the high rates of incidence and mortality of lung cancer, the A549 strain was chosen to continue the experiments.

**Table 2:** IC_50_ (µg/mL) values for *M. splendens* essential oil in cancer cell lines

Cells	*M. splendens* essential oil
	IC_50_ values (µg/mL)
A549	20,14
	16,46–24,64
B16-F10	17,76
	9,48–33,26
THP-1	5,37
	2,17–13,30

Data are presented as IC_50_ values in µg/mL and their 95% confidence interval obtained by non-linear regression from three independent experiments performed quadruplicate by MTT assay after 24 h of incubation

### *M. splendens* essential oil reduces the ability of A549 lung cancer cells to form colonies

**Fig. 1:**
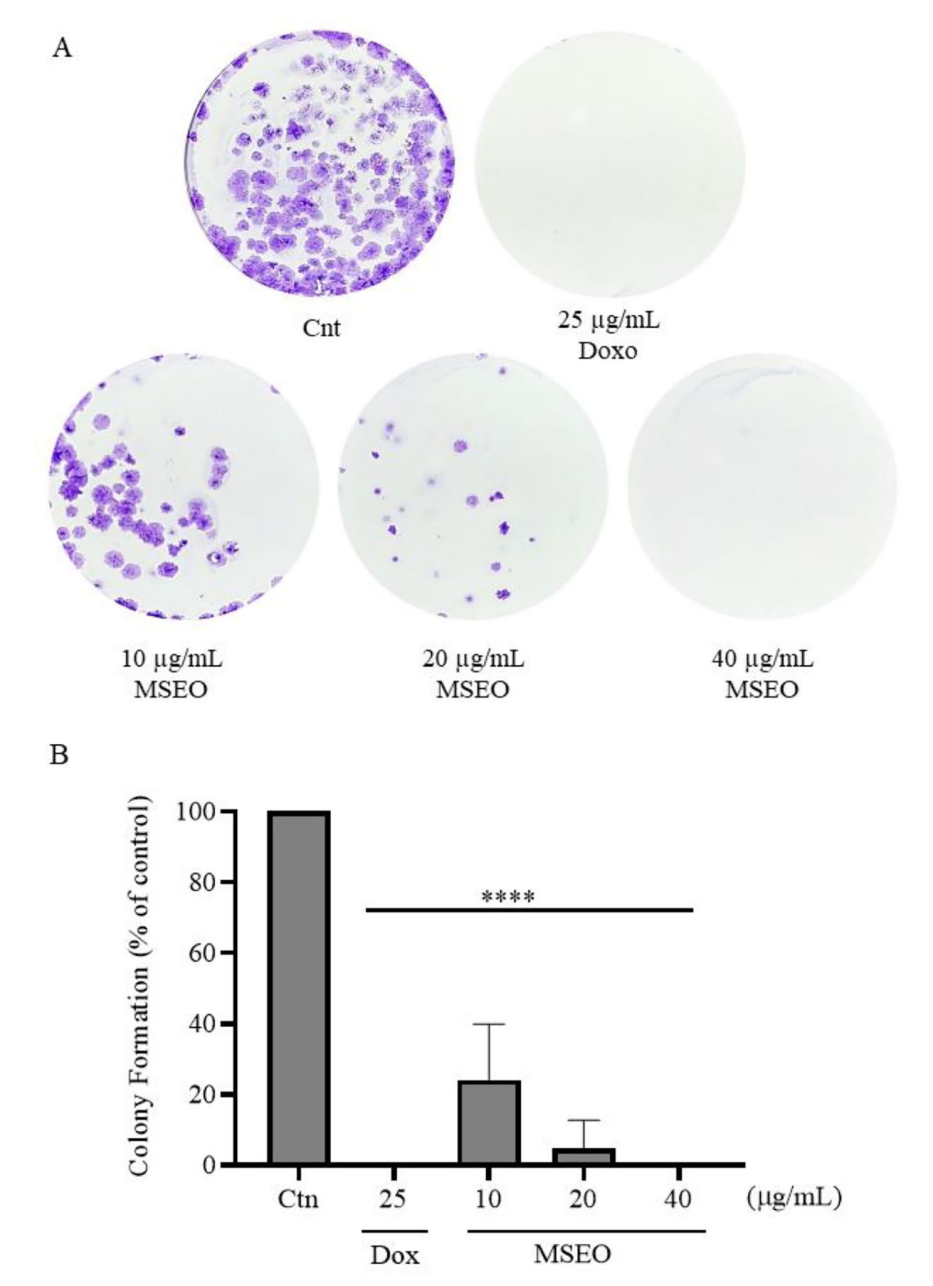
Inhibitory effect of the *M. splendens* EO on the formation of A549 lung cancer cells colonies. (**A**) Colonies formed after treatment with increasing concentrations (10, 20 and 40 µg/mL) for 24 h of *M. splendens* EO and 10 days of growth. (**B**) Percentage of colony formation after treatment compared to control cells. Doxorubicin (25 µg/mL) was used as a positive control. Data show are mean ± SD obtained from three independent experiments. Statistical differences, compared to untreated control cells, were assessed by a one-way ANOVA with Dunnett post-test (****) p < 0.0001

To assess whether EO has the ability to inhibit the formation of colonies of A549 cells, a clonogenic assay was performed. Cells were treated for 24 h with EO at concentrations of 10, 20 and 40 µg/mL, which correspond to the values of 0.5xIC_50_, 1xIC_50_ and 2xIC_50_, respectively. After the treatment, the cells were kept growing for 10 days, and then the analyzes were performed. The results obtained are shown in Fig. 1. A significant reduction in the number of colonies (p < 0.0001) was observed in the treatment with EO at all concentrations tested, compared to the negative control. Treatment with Doxorubicin (25 µg/mL) also caused a significant reduction (p < 0.0001) compared to untreated cells. The data are represented in Fig. 1B.

### *M. splendens* essential oil inhibits the migratory and invasive ability of A549 cells

**Fig. 2:**
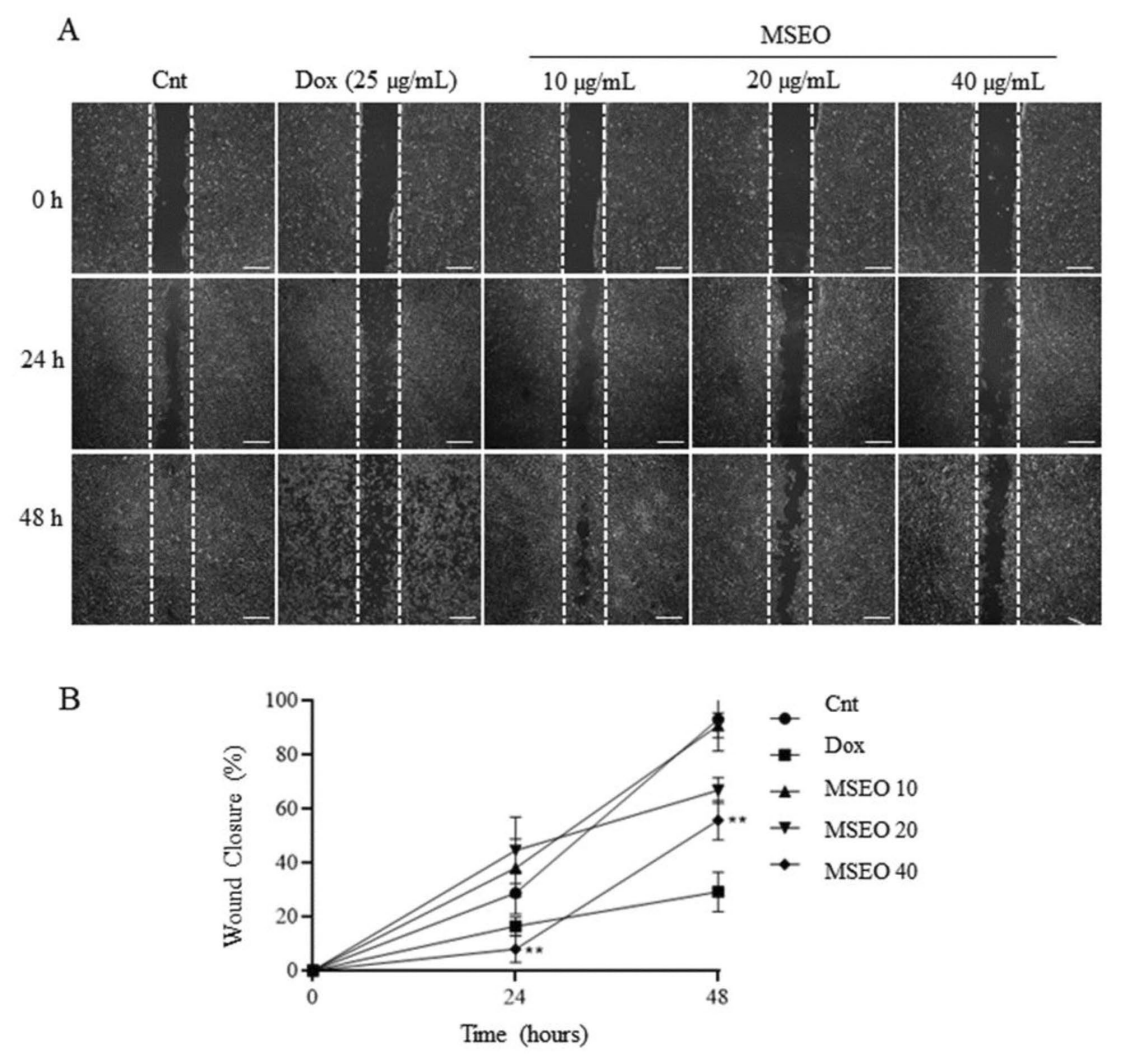
Inhibitory effect of the *M. splendens* EO on the migratory and invasive ability of A549 cells. (**A**) Representative photograph of the Wound Healing assay after treatment with increasing concentrations (10, 20 and 40 µg/mL) for 0, 24 and 48 h of *M. splendens* EO. (**B**) Percentage wound closure after treatment compared to control cells. Doxorubicin (25 µg/mL) was used as a positive control. Data show are mean ± SD obtained from three independent experiments. Statistical differences, compared to untreated control cells, were assessed by a Two-way ANOVA with Dunnett post-test (**) p < 0.05

The migration assay, using the Wound Healing technique, was performed to verify the effects of EO treatment on the migratory capacity of A549 cells. EO concentrations of 10, 20 and 40 µg/mL were used, and the analyzes were performed at 0, 24 and 48 h. Figure 2 shows the wound closure process in the cell monolayer, with representative images of the wells. According to the data obtained, it was possible to observe a significant effect on the migratory capacity (Fig. 2) of A549 cells at a concentration of 40 µg/mL after 24 h of treatment.

### *M. splendens* essential oil induces apoptosis in A549 cells

**Fig. 3:**
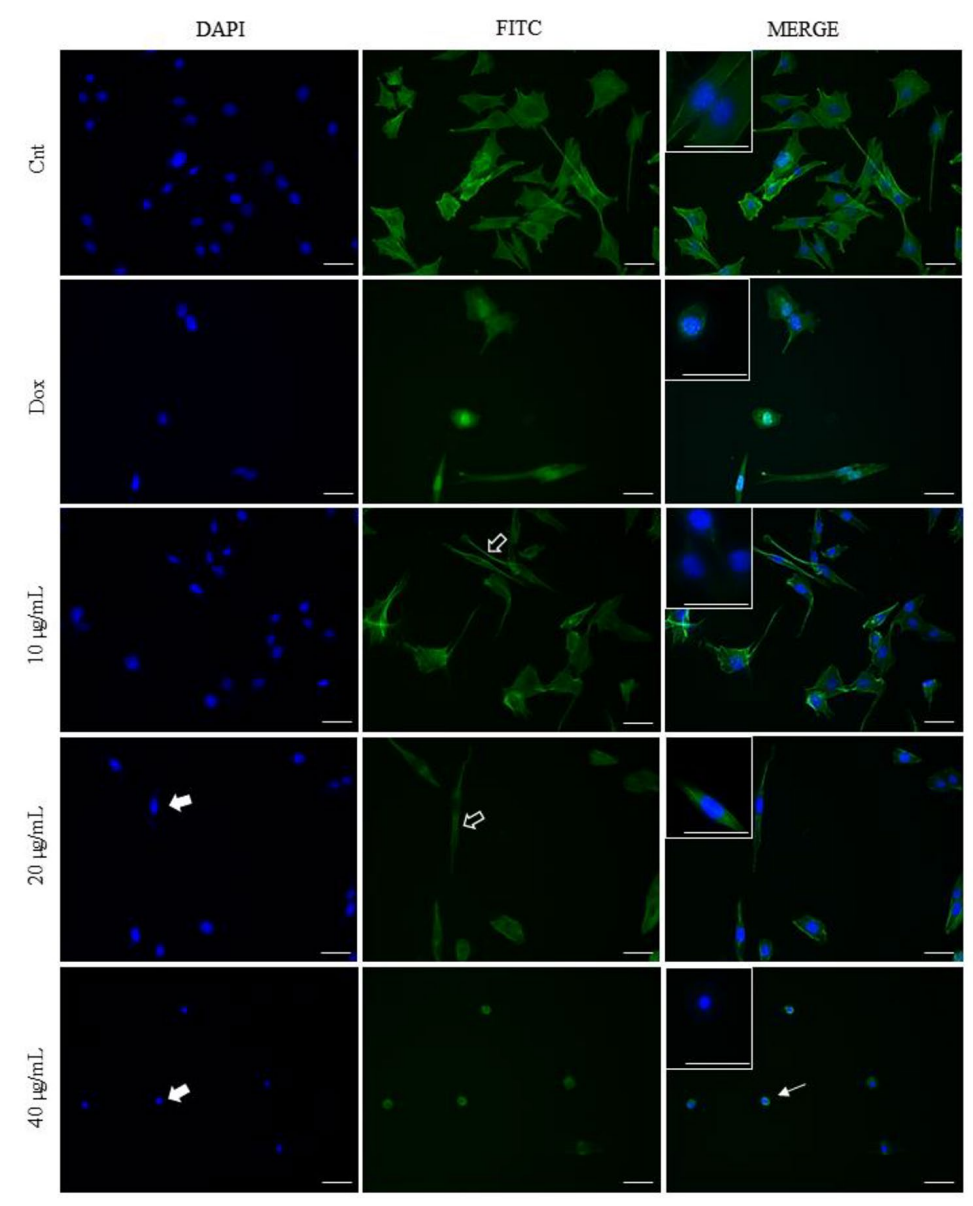
Morphological changes in the cytoskeleton and nucleus of A549 cells, observed with DAPI and Phalloidin/FITC staining, after treatment for 24 h with *M. splendens* EO at concentrations of 10, 20 and 40 µg/mL. Doxorubicin (25 µg/mL) was used as a positive control. Filled arrows represent chromatin condensation and DNA fragmentation; hollow arrows represent reduction in cytoplasmic volume and thin arrows, rounding and cell shrinkage. Bar: 20 μm

The apoptotic morphological changes in the nucleus and cytoplasm of A549 cells after 24 h of treatment with EO were evaluated using DAPI and Phalloidin/FITC dyes. Figure 3 shows the morphological changes caused by increasing concentrations of EO. According to the data presented, a reduction in cytoplasmic volume was observed in A549 cells at a concentration of 10 µg/mL of EO. At a concentration of 20 µg/mL, in addition to the reduction in cytoplasmic volume, cells with greater accumulation of DAPI in the nucleus were also observed, indicating DNA fragmentation and chromatin condensation. In cells treated at a concentration of 40 µg/mL, rounding and cytoplasmic and nuclear shrinkage were observed, in addition to chromatin condensation and DNA fragmentation. Treatment with Doxorubicin caused important cellular changes such as reduced cytoplasmic volume, chromatin condensation and DNA fragmentation. Overall, the most observed changes after treatment with EO concentrations were: decrease in cell number, cell rounding and shrinkage, and reduction of cytoplasmic volume, characteristics of programmed cell death induction. For the same treatment time, the observed changes were more intense as the EO concentration was increased.

## Discussion

In this study, we demonstrated the cytotoxicity of essential oil from *M. splendens* leaves against the human lung adenocarcinoma cell line (A549). A previous study investigated the chemical characterization and biological activities, including cytotoxicity, of the EO from the leaves of *M. splendens* collected in Amazonian Ecuador. The authors found trans-nerolidol and α-bisabolol as the major EO compounds [9]. Interestingly, in the present study we observed a different chemical composition of the EO, in which the major compounds found were bicyclogermacrene, E-caryophyllene and germacrene D. This difference in the chemical composition of the *M. splendens* EOs may have occurred mainly due to the variation of the place of collection of the leaves, since the samples used in the present study were collected in the northeast of Brazil. Previous studies have reported that factors such as high genetic diversity can influence the variability of essential oils from species of the Myrtaceae family [17]. Despite the difference between the major *M. splendens* EO compounds found in our study and that of Scalvenzi et al. (2017), the chemical composition of the EO evaluated in both studies is compatible with regard to the predominance of sesquiterpenes.

Previous studies suggest that for an EO to be considered promising for cancer drug development, its IC_50_ values need to be less than 30 µg/mL [18]. In the present study, we observed that EO caused cytotoxicity in THP-1 (acute monocytic leukemia), B16-F10 (melanoma) and A549 (human lung adenocarcinoma) cell lines, with IC_50_ values less than 21 µg/mL. A study by Mohamed et al. (2018) reported cytotoxic activity in A549 cells treated with essential oil of *Pistacia lentiscus* L., which contained sesquiterpene germacrene D as one of the main compounds [19]. Another study reported that germacrene D and bicyclogermacrene, in synergism, were responsible for the high cytotoxic activity of essential oil from *Porcelia macrocarpa* leaves against leukemic cells (HL-60) [20]. In research carried out by The et al. (2021), the cytotoxic potential of the essential oil of the leaves of *Polyalthia suberosa* was evaluated, whose major constituents were bicyclogermacrene, E-caryophyllene and β-pinene. The results obtained showed cytotoxic activity against tumor cell lines of hepatocellular carcinoma (HepG2), breast cancer (MCF-7) and human lung adenocarcinoma (A549) [21]. These studies demonstrate that the sesquiterpenes bicyclogermacrene, E-caryophyllene and germacrene D are compounds that have antitumor action. The fact that these are the major constituents found in EO suggests that they may be the main components responsible for the antitumor action of *M. splendens* EO. However, studies are needed to investigate the action of these isolated compounds, for a definition of their antitumor activity.

In addition, EO was shown to significantly reduce (p < 0.05) the percentage of A549 cell colonies at all concentrations evaluated. Our results were similar to those obtained in the study by Dahham et al. (2015), who evaluated the anticlonogenic activity of sesquiterpene β-caryophyllene in colon carcinoma cells (HCT 116) [22]. A study by Toyang et al. (2013) reported the anticlonogenic activity of two sesquiterpenes isolated from *Vernonia guineensis* leaves, Vernopicrin and Vernomelitensin, which reduced the number of colonies in the prostate adenocarcinoma cell line (PC-3), during the nine-day exposure period, in a period similar to that used in the present study [23].

Among the mechanisms involved in the development and establishment of cancer is the process of tumor cell migration. During this process, tumor cells migrate from the primary site to a secondary organ, being a critical process of invasion, which allows the adaptation of primary tumors to metastasis [24, 25]. Our results indicate that EO causes a significant reduction in the migration capacity of A549 cells at the highest concentration (40 µg/mL), after 24 h of treatment. Studies show that essential oils have the ability to reduce tumor cell migration [26, 27]. The study by Chen et al. (2018), the essential oil of *Eupatorium adenophorum* Spreng., consisting mainly of sesquiterpenes, reduced the migration of hepatocellular carcinoma (HepG2) cells [28].

Furthermore, *M. splendens* EO was able to induce apoptosis in A549 cells, a cell death mechanism observed through changes in the nucleus and cytoplasm of A549 cells [29]. All concentrations promoted a reduction in cytoplasmic volume, while DNA fragmentation and chromatin condensation, evidenced by the accumulation of DAPI present in the cell nucleus, were only observed from a concentration of 20 µg/mL. This apoptotic effect was also observed in the study by Pereira et al. (2017), when evaluating the essential oil of *Baccharis milleflora* (Less.) DC against non-Hodgkin’s lymphoma (Raji) cells. The authors found bicyclogermacrene, germacrene D, E-caryophyllene and α-humulene as major compounds in the essential oil of *Baccharis milleflora* (Less.) DC [30].

## Conclusion

This study showed that *M. splendens* EO has a cytotoxic effect on A549 lung cancer cells. The EO was able to reduce colony formation and cell migration, in addition to inducing death by apoptosis. Based on GC/MS, the study demonstrated that the EO is rich in sesquiterpenes. Considering the biological activities of sesquiterpenes, including antitumor activity, the cytotoxic effect of EO on A549 cells could have been attributed to these compounds. Thus, additional research is needed to investigate the biochemical mechanisms of *M. splendens* EO and its isolated compounds. Such findings may be useful to guide searches for potential new antitumor agents.

## Data Availability

The datasets generated and/or analyzed in this study are available from the corresponding author on reasonable request.

## Abbreviations

A549: Human Lung Carcinoma Epithelial Cell Line
B16-F10: Melanoma Cell Line
CDK: Cyclin Dependent Kinases
DAPI: 6-Diamidine-2-phenylindole dihydrochloride
DMSO: Dimethylsulfoxide
DOX: Doxorubicin
EDTA: Ethylenediaminetetraacetic acid
EO: Essential Oil
FBS: Fetal Bovine Serum
GC-MS: Gas Chromatography Coupled with Mass Spectrometry
IC_50_: Inhibitory concentration for 50% of cells
INCA: José Alencar da Silva National Cancer Institute
MCF-7: Adenocarcinoma Cell Line
MSEO: *Myrcia splendens* Essential Oil
PHALLOIDIN/FITC: Fluorescein Isothiocyanate
PSB: Phosphate Saline Buffer
THP-1: Leukemia Cell Line
WHO: World Health Organization
